# Effect of Developmental Stages on Genes Involved in Middle and Downstream Pathway of Volatile Terpene Biosynthesis in Rose Petals

**DOI:** 10.3390/genes13071177

**Published:** 2022-06-30

**Authors:** Ying Kong, Huan Wang, Lixin Lang, Xiaoying Dou, Jinrong Bai

**Affiliations:** 1 Institute of Radiation Technology, Beijing Academy of Science and Technology, Beijing 100875, China; kongying@brc.ac.cn (Y.K.); wanghuan@brc.ac.cn (H.W.); langlixin@brc.ac.cn (L.L.); douxiaoying@brc.ac.cn (X.D.); 2 Key Lab of Beam Technology and Material Modification of Ministry of Education, College of Nuclear Science and Technology, Beijing Normal University, Beijing 100875, China; 3 School of Landscape Architecture, Beijing Forestry University, Beijing 100083, China

**Keywords:** *Rosa*, terpene synthase, *trans*-prenyltransferases, NUDIX, farnesene synthase

## Abstract

Terpenoids are economically and ecologically important compounds, and they are vital constituents in rose flower fragrance and rose essential oil. The terpene synthase genes (TPSs), *trans*-prenyltransferases genes (TPTs), *NUDX1* are involved in middle and downstream pathway of volatile terpene biosynthesis in rose flowers. We identified 7 complete *RcTPT*s, 49 complete *RcTPS*s, and 9 *RcNUDX1* genes in the genome of *Rosa* *chinensis*. During the flower opening process of butterfly rose (*Rosa* *chinensis* ‘Mutabilis’, MU), nine *RcTPSs* expressed in the petals of opening MU flowers exhibited two main expression trends, namely high and low, in old and fresh petals. Five short-chain petal-expressed *RcTPTs* showed expression patterns corresponding to *RcTPS*s. Analysis of differential volatile terpenes and differential expressed genes indicated that higher emission of geraniol from old MU petals might be related to the *RcGPPS* expression. Comprehensive analysis of volatile emission, sequence structure, micro-synteny and gene expression suggested that *RcTPS18* may encode (*E*,*E*)-α-farnesene synthase. These findings may be useful for elucidating the molecular mechanism of terpenoid metabolism in rose and are vital for future studies on terpene regulation.

## 1. Introduction

Volatile terpenoids constitute the largest class of plant volatile compounds [1]. All plant organs, such as leaves, branches, flowers, and roots, can emit terpene volatiles to ensure healthy plant growth [2]. Petals are the main release parts of floral fragrance in many plants, such as rose (*Rosa* spp.) [3]. The role of volatile terpenoids released from flowers is to attract pollinators, and to defense against biotic and abiotic stresses [4]. For example, geraniol has certain antibiotic activity and can be detected with high response by the honeybees’ antennae [5]. The β-ocimene and linalool were common attracting compounds for pollinators and has antibacterial effect [6,7]. (*E*)-α-farnesene released from the flowers of *Brassica rapa* showed attraction of bees instead of butterflies [8], and (*E*)-β-caryophyllene is beneficial for plant fitness and functions in defense against pathogenic bacteria [9].

In most plants, the volatile terpenoids are constructed from two C_5_ precursors, namely isopentenyl diphosphate (IPP) and its isomer dimethylallyl diphosphate (DMAPP), which are produced through either the methylerithritol phosphate pathway (MEP) in the chloroplast or the mevalonate pathway (MVA) in the cytosol [4,10]. Then, the IPP and DMAPP units are condensed by prenyltransferases (PTs, also referred to as isoprenyl diphosphate synthases or prenyl diphosphate synthases) to form direct terpene precursors, such as geranyl diphosphate (C_10_, GPP), farnesyl diphosphate (C_15_, FPP), or geranylgeranyl diphosphate (C_20_, GGPP) [11]. Subsequently, terpene synthase (TPS), which is the primary enzyme in the terpenoid biosynthetic pathways, converts the precursors into various terpene products, such as monoterpene (C_10_), sesquiterpene (C_15_), and diterpene (C_20_) [12]. These products can undergo further modifications under the action of various enzymes, such as dehydrogenases, methyltransferases, acyltransferases, and glycosyltransferases to form highly diverse metabolites [13,14].

Rose is widely cultivated as a garden plant for the cut-flower industry, and floral fragrance is a vital characteristic of ornamental roses [14,15]. The terpenoid volatiles in the floral rose scent are mainly monoterpenes and sesquiterpenes, as well as their derivatives. Compared with moderately and less fragrant rose cultivars, the very fragrant cultivars produced a certain amount of monoterpenoids, including geraniol, citronellol, and nerol, all three of which have roselike fragrances [16]. In addition, the aldehyde and acetate ester derivatives of these three compounds were also produced [16,17]. Other monoterpenoids including α-pinene, β-pinene, limonene, and linalool are emitted at low levels in rose floral scent [17]. Sesquiterpenes, including germacrene D, δ-cadinene, α-copaene, α-cubenene, β-cubenene, β-elemene, and β-caryophyllene, are emitted from rose flowers, whereas germacrene D was highly released from some rose cultivars [17,18,19,20].

Only several genes involved in the biosynthesis of rose volatile terpenes have been identified. The first TPS gene cloned in rose was germacrene D synthase (*RhGDS*), which catalyzes the substrate FPP to germacrene D as a unique product [20]. Three TPS genes, namely *RcLINS*, *RcLIN-NERS1*, and *RcLIN-NERS2*, have been characterized, and the expression levels of these genes were low in rose petals [21]. *RcLINS* belonging to the TPS-b subfamily is responsible for the presence of a small amount of (*3R*)-(-)-linalool in rose scent. The bifunctional *RcLIN-NERS1* and *RcLIN-NERS2*, belonging to the TPS-g subfamily, produce (*3S*)-(+)-linalool and nerolidol when incubated with GPP and FPP, respectively, whereas *RcLIN-NERS3* has been identified as a pseudogene. Moreover, a novel TPS-independent pathway for monoterpene biosynthesis was described in rose [22]. An enzyme of the Nudix hydrolase family (RhNUDX1) localized in the cytoplasm was reported to be involved in geraniol biosynthesis. Another study showed that *RwNUDX1-2* was involved in the biosynthesis of a group of sesquiterpenoids [23]. However, no *trans*-prenyltransferases (TPT) gene has been characterized in *Rosa* plants.

*Rosa chinensis* ‘Mutabilis’ (butterfly rose, MU), with single petals, is a fragrant ancient Chinese rose cultivar. Some monoterpene alcohols were released from MU flowers, including geraniol, nerol, and linalool, and the monoterpene contents in MU exhibit a significant increase from unopened buds to floral maturity and further accumulation during senescence [18,24]. The butterfly rose is a good material to study the metabolism of floral volatile terpenoids.

Although *cis*-prenyltransferases (CPTs) that initially predicted to synthesize long-chain isoprenyl diphosphates were involved in terpenoids biosynthesis pathway, none of the three *RcCPT* genes were expressed in MU petals on our pre-study so we focused on the *trans*-prenyltransferases (TPT) genes [25,26]. A very recently published article identified the 49 TPS genes in *R*. *chinensis*, but the results included three pseudogenes and three missing full-length genes [27]. In this study, a genome-wide identification of the genes involved in middle and downstream pathway of volatile terpene biosynthesis in *R*. *chinensis* was conducted, including TPS genes, TPT genes and *RcNUDX1* genes. RNA sequencing (RNA-seq) was performed to investigate the expression patterns of these terpene-related genes during different flower development stage. Furthermore, the differential volatile terpenes and differential expressed genes (DEGs) were analyzed to elucidate the potential functions of DEGs. These results may be useful for decoding the genes involved in terpene biosynthesis pathways, which provide insights for the manipulation of genetic engineering in rose and other plants.

## 2. Materials and Methods

### 2.1. Identification and Phylogenetic Analysis of RcTPT Gene Family

The complete rose (*R*. *chinensis* ‘Old Blush’) genome sequence was obtained from the official website (https://lipm-browsers.toulouse.inra.fr/pub/RchiOBHm-V2/, accessed on 4 May 2022). In total, 26 TPT protein sequences of *Arabidopsis thaliana* and tomato (*Solanum lycopersicum*) (Table S1) were used as a query for BLASTP search with default parameters. A Hidden Markov Model (HMM) search were also conducted by using polyprenyl synthase domain (PF00348) [28], with an E-value < 0.001. The BLASTP and HMM search results were integrated to identify candidate TPT genes. Then, a maximum likelihood phylogenetic tree was constructed using RAxML online platform (https://raxml-ng.vital-it.ch, accessed on 13 June 2022). Signal peptides were predicted using TargetP 2.0 (https://services.healthtech.dtu.dk/service.php?TargetP-2.0, accessed on 13 June 2022) and LOCALIZER (https://localizer.csiro.au/, accessed on 13 June 2022) online platform [13,29].

### 2.2. Re-Identification and Sequence Analysis of the RcTPS Gene Family

Two HMM profiles (PF03936 and PF01397) were used as a query to search the rose genome [30], with an E-value < 0.001. All candidate TPS genes were aligned using MAFFT v7.475 [31] before manually figuring out the conserved regions. The genes with all conserved regions and expected gene structure were classified as complete *RcTPS* genes, whereas those with incomplete or mutated conserved regions were classified as partial/pseudo (TPS-p) genes.

### 2.3. Chromosomal Localization and Collinearity Analyses

Nine homologous *RcNUDX1* genes were identified as described by Sun et al. [23]. The *RcTPTs*, *RcTPSs,* and *RcNUDX1* genes of *R*. *chinensis* were mapped on the chromosomes according to their positions in the annotated genome documents by using TBtools v1.0 [32]. The tandemly duplicated genes were confirmed based on three criteria: (a) length of alignable sequence covered >70% of the longer gene; (b) similarity of aligned regions >70%; (c) close chromosome location (<100 kb) and few separated genes (≤5) [33]. 

Collinearity analysis within *R*. *chinensis* was conducted, and segmentally duplicated genes were identified in the collinear segments. The whole-genome sequences and annotation documents of peach (*Prunus persica*), strawberry (*Fragaria vesca*), and *R. rugosa* were downloaded from The Genome Database for Rosaceae [34]. The whole-genome sequences and annotation documents of grapevine (*Vitis vinifera*) were downloaded from Phytozome. The interspecific collinearity analysis between *R*. *chinensis* and these plants was performed using TBtools software to determine the interspecies collinear relationships among orthologous TPS and TPT genes [32].

### 2.4. Plant Materials

**Figure 1:**
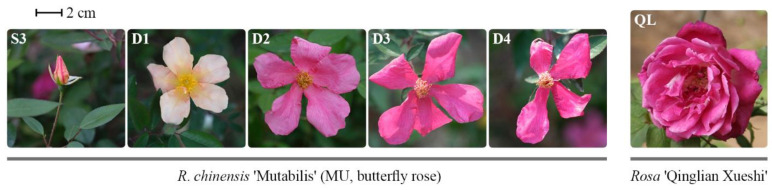
Photos of butterfly rose (*R. chinensis* ‘Mutabilis’) at different floral developmental stages and *Rosa* ‘Qinglian Xueshi’. S3: bud about to open; D1: first day of anthesis; D2: second day of anthesis; D3: third day of anthesis; D4: fourth day of anthesis.

Two Chinese old rose cultivars, butterfly rose (*R. chinensis* ‘Mutabilis’, MU) and *Rosa* ‘Qinglian Xueshi’ (QL), were collected from Kunming Yang Chinese Rose Gardening Co., Ltd. (Kunming, China), and planted in the germplasm garden of our institute under open field conditions (116°43′ N, 40°16′ E) for 2–3 years. According to our observation, the MU flowering process lasts approximately 4 days. Different floral developmental stages of MU flowers, namely bud about to open (S3), first day of anthesis (D1), second day of anthesis (D2), third day of anthesis (D3), and fourth day of anthesis (D4), were analyzed (Figure 1). 

The upper half of rose petal without additional anthocyanin coloration in the abaxial surface was sampled from different individuals at 8:00 a.m.–9:00 a.m. on sunny days and frozen in liquid nitrogen, and the samples were stored at −80 °C. Three biological replicates collected on different days were used as samples. More than 60 flowers were collected at D1 and D3 stages, and more than 30 flowers were collected at S3, D2, and D4 stages (approximately 0.13 g per flower). 

### 2.5. RNA-Seq Analysis

Then, five butterfly rose samples at different developmental stages were used for RNA-seq. The samples stored at −80 °C were sent to Guangzhou Gene Denovo Biological Technology Co., Ltd. (Guangzhou, China) to perform RNA isolation, RNA-seq library preparation, and sequencing [35]. The libraries of three biological replicates were prepared independently. After removing low-quality reads, the clean reads were mapped to the *R. chinensis* reference genome (https://lipm-browsers.toulouse.inra.fr/pub/RchiOBHm-V2/, accessed on 4 May 2022), and the FPKM (fragments per kilobase million) value was used to determine the gene expression levels. The raw sequence data reported in this paper have been deposited in the Genome Sequence Archive [36] in the National Genomics Data Center [37], China National Center for Bioinformation/Beijing Institute of Genomics, Chinese Academy of Sciences (GSA: CRA006521) that are publicly accessible at https://ngdc.cncb.ac.cn/gsa (accessed on 4 May 2022).

### 2.6. Volatile Sampling and Gas Chromatography–Mass Spectrometry (GC-MS) Analysis

The fresh petals (D1) and old petals (D3) of butterfly rose were selected for floral volatile analysis, whereas the fully bloomed QL flowers collected at the same time were used as control. After the samples were grounded into powder in liquid nitrogen, 1 g of the powder was transferred immediately to a 20 mL headspace vial (Agilent, Palo Alto, CA, USA) containing NaCl-saturated solution to inhibit any enzymatic reaction [38,39]. The vials were sealed using crimp-top caps with TFE-silicone headspace septa (Agilent) and heated at 100 °C for 5 min. Then, 120 µm divinylbenzene, carboxen, or polydimethylsilioxan fiber (Agilent) was exposed to the sample headspace for 15 min at 100 °C. 

VOC identification and quantification were conducted using an Agilent Model 8890 GC and a 5977B mass spectrometer (Agilent, Palo Alto, CA, USA) equipped with a DB-5MS (30 m × 0.25 mm × 0.25 μm) capillary column. After sampling, desorption was performed at 250 °C for 5 min in the split-less mode of the GC apparatus. Helium was used as the carrier gas at a linear velocity of 1.2 mL/min. The oven temperature was programmed from 40 °C (3.5 min), increasing at 10 °C/min to 100 °C, at 7 °C/min to 180 °C, at 25 °C/min to 280 °C, and hold for 5 min. Other GC-MS analytical conditions used were as described by Gong et al. [39]. Volatile compounds were identified by comparing the mass spectra with the data system library (MWGC or NIST) and retention index.

### 2.7. qRT-PCR Analysis

Total RNA was extracted using the OmniPlant RNA Kit (DNase I) (CoWin Biosciences, Taizhou, China). The first strand of cDNA was synthesized from 15 μL of total RNA by using MonScript™ RTIII All-in-One Mix with dsDNase (Monad Biotechnology Co., Ltd., Wuhan, China). *RhUBI2* (JK618216) and *Actin* were used as internal controls [40]. Primers were designed using Primer Premier 5.0 software (Premier Biosoft International, Palo Alto, CA, USA) and synthesized by Sangon Biotech (Shanghai, China) (Table S2). qRT-PCR was performed on a ABI 7500 FAST DX Real-Time PCR instrument (Thermo Fisher Scientific, Inc., Waltham, USA). Each reaction was conducted in a 20 μL mixture containing 10 μL of 2× Universal Blue SYBR Green qPCR Master Mix (Wuhan Servicebio Biotechnology Co., Ltd., Wuhan, China), 7.4 μL of RNase-free H_2_O, 1 μL of cDNA, 0.8 μL of forward primer, and 0.8 μL of reverse primer. The PCR machine was programmed as follows: PCR initial activation step for 2 min at 95 °C, followed by 40 cycles at 95 °C for 5 s and 60 °C for 30 s. The relative gene expression was calculated using the 2^−ΔΔCT^ method [41].

### 2.8. Data Analyses 

Differential expression analysis between D1 and D3 samples was performed using DeSeq2 in OmicShare tools (www.omicshare.com/tools, accessed on 4 May 2022), with a Q-value threshold of 0.05. Heatmap were performed using the ComplexHeatmap and pheatmap package [42]. One-way analysis of variance (ANOVA) followed by Waller-Duncan post-hoc test was applied to examine the data significant levels among groups by SPSS 23 (SPSS Inc., Chicago, IL, USA). The Student’s test was performed to determine the difference between two groups by Microsoft Excel 2019 (Seattle, WA, USA). After Pareto scaling the volatile emission abundance by SIMCA software (V14.1, MKS Data Analytics Solutions, Umea, Sweden), the variable importance in projection (VIP) of volatile terpenoids was calculated for the subsequent screening of differential compounds [43].

## 3. Results

### 3.1. Identification of RcTPT Genes

**Figure 2:**
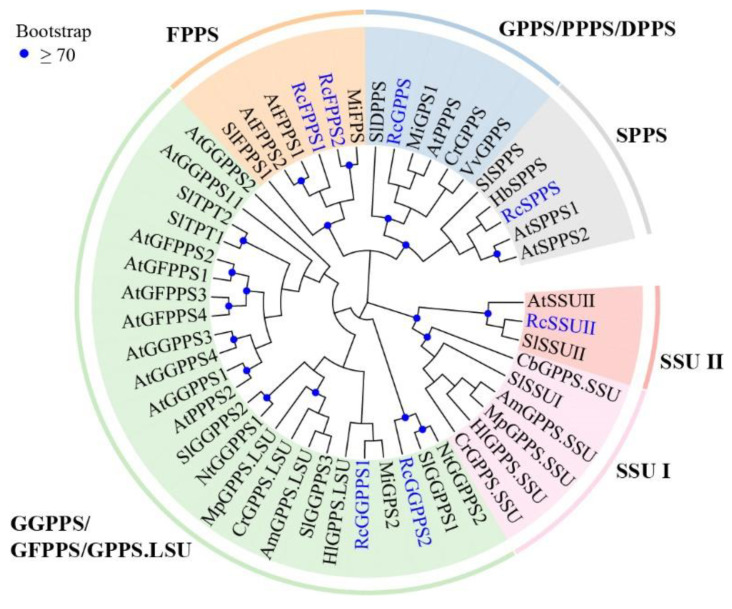
A maximum likelihood phylogenetic tree was constructed by aligning the amino acid sequences of seven RcTPTs and other characterized *trans*-prenyltransferases in eudicots. RcTPTs are highlighted in blue. The sequences used in this analysis are listed in Table S1.

Based on the BLASTp and HMMER search results, 17 candidate TPT genes were originally obtained from the genome of *R*. *chinensis*. After aligning the candidate sequences by MAFFT software, seven genes comprising five typical domains were identified full-length *RcTPT* genes, encoding polypeptides ranged from 329 to 426 amino acids (Figure S1). A maximum likelihood phylogenetic tree was constructed using protein sequences of seven complete RcTPTs and other characterized TPTs in eudicots to explore the evolutionary relationship. Phylogenetic analysis grouped RcTPTs into two RcFPPS, two RcGGPPS, one RcSSUII, one RcGPPS, and one RcSPPS (Figure 2). These genes were renamed based on subgroup, including six putative short-chain TPTs and one putative long-chain TPT (Table 1). The other 10 genes encoding shorter proteins were categorized as partial genes as they contained partial TPT domains (Table S3).

### 3.2. Re-Identification of RcTPS Genes

The present study originally obtained 80 nonredundant candidate gene models corresponding to PF01397 or PF03936. After removing the sequences that did not contain the typical TPS domains, a total of 74 putative TPS genes were identified, 49 of which were predicted to encode functional TPS enzymes. Each complete *RcTPS* gene had an open reading frame of expected size and organization and contained typical TPS domains comprising either the Mg^2+^ binding (DDxxD/E) and NSE/DTE regions or the DxDD motif (Figure S2). The remaining 25 TPS genes were categorized as partial/pseudo genes as they contained partial or mutant TPS domains (Table S3). The genes were then renamed based on their locations for better understanding (Table 2). The candidate TPS genes identified were *RcGDS* (MG673512, *RcTPS30*), *RcLINS* (MG673509, *RcTPS-p9* + *RcTPS-p10*), *RcLIN-NERS1* (MG673510, *RcTPS20*), *RcLIN-NERS2* (MG673511, *RcTPS22*), and *RcLIN-NERS3* (MG673515, *RcTPS23*). Phylogenetic analysis separated RcTPSs into five groups, including 32 TPS-a, 8 TPS-b, 4 TPS-g, 3 TPS-e/f, and 2 TPS-c genes (Figure S2).

### 3.3. Chromosomal Localization and Gene Duplication

**Figure 3:**
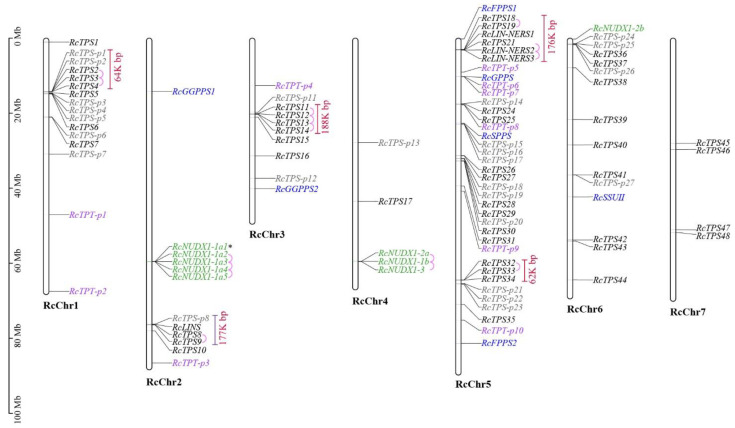
Chromosomal distribution of *RcTPSs*, *RcTPTs*, and *RcNUDX1* genes in the *R. chinensis* genome. The corresponding IDs of *RcNUDX1* genes are listed in Table S4. Black letters represent putative complete TPS genes, blue letters represent complete *trans*-prenyltransferase (TPT) genes, gray letters represent putative partial/pseudo TPS (*TPS-p*) genes, purple letters represent partial TPT (TPT-p) genes, and green letters represent *RcNUDX1* genes. The tandemly duplicated genes are indicated in pink lines, and gene clusters are indicated in red lines. *Asterisk indicates a stop codon is interrupting the open reading frame of this sequence [23].

The complete and partial/pseudo *RcTPSs* and *RcTPTs*, along with nine *RcNUDX1* genes were mapped to the seven chromosomes of the *R. chinensis* (Figure 3). RcChr5 contained the largest number of TPT and TPS genes, including 18 *RcTPSs*, 10 *RcTPS-p* genes, 4 *RcTPTs* and 6 *RcTPT-p* genes, which suggest the multiple duplication and recombination events on this chromosome [44]. Most TPS-p genes were distributed near the putative full-length TPS genes. Six tandemly duplicated TPS genes were present in the *R. chinensis* genome, which occurred in the TPS-a, -b, and -g subfamilies, forming five gene clusters. Some of the *RcTPS* genes were localized in the vicinity of RcTPT genes, which indicated that some *RcTPS* and *RcTPT* genes probably evolved together through genomic duplication [30]. Additionally, no segmentally duplicated *RcTPS* or *RcTPT* genes were detected in the *R. chinensis* genome.

### 3.4. Collinearity Analysis of RcTPT and RcTPS Genes

**Figure 4:**
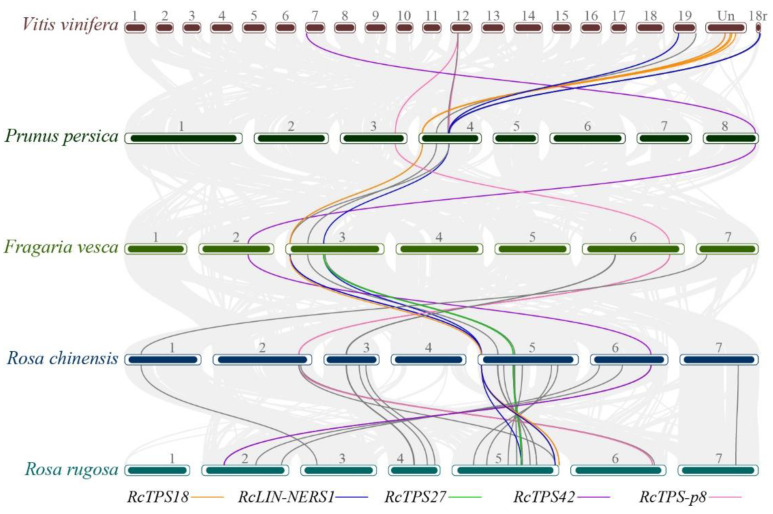
Collinearity analysis of TPS genes among *R. chinensis* and other representative plant species. Gray lines in the background indicate the collinear blocks between rose and other plant genomes. The color and black lines highlight the syntenic TPS gene pairs.

The comparative collinearity maps of *R. chinensis* associated with other representative species were constructed to further infer the phylogenetic mechanisms of the TPS and TPT gene family. Four *RcTPS* genes, namely *RcTPS18*, *RcLIN-NERS1*, *RcTPS42*, and *RcTPS-p8*, exhibited genomic shuffling across the Rosaceae species and grapevine, indicating that these genes were derived probably from the ancestors of dicotyledonous plants (Figure 4). Unlike the other three genes with only one collinear gene pair in each plant genome, *RcLIN-NERS1* had three collinear gene pairs in the *R. rugosa* genome and strawberry genome. *RcLIN-NERS1* and its two collinear genes in strawberry (*FaNES1* and *FaNES2*) are bifunctional terpene synthase that can efficiently convert GPP and FPP into linalool and nerolidol, respectively [21,45]. This indicates that collinear TPS genes may exhibit similar catalytic function in relative plants. *RcTPS27* (TPS-a) and *RcLIN-NERS1* (TPS-b) in *R. chinensis* shared the same collinear TPS genes in *R. rugosa* and strawberry, indicating that TPS-a (*RcTPS27*) is probably derived from TPS-g. Unlike the RcTPS gene family, there are six RcTPT genes exhibited genomic shuffling across the four Rosaceae species, suggesting the TPT gene family is evolutionarily conserved in Rosaceae (Figure S3).

### 3.5. Effect of Developmental Stages on RcTPS Expression

**Figure 5:**
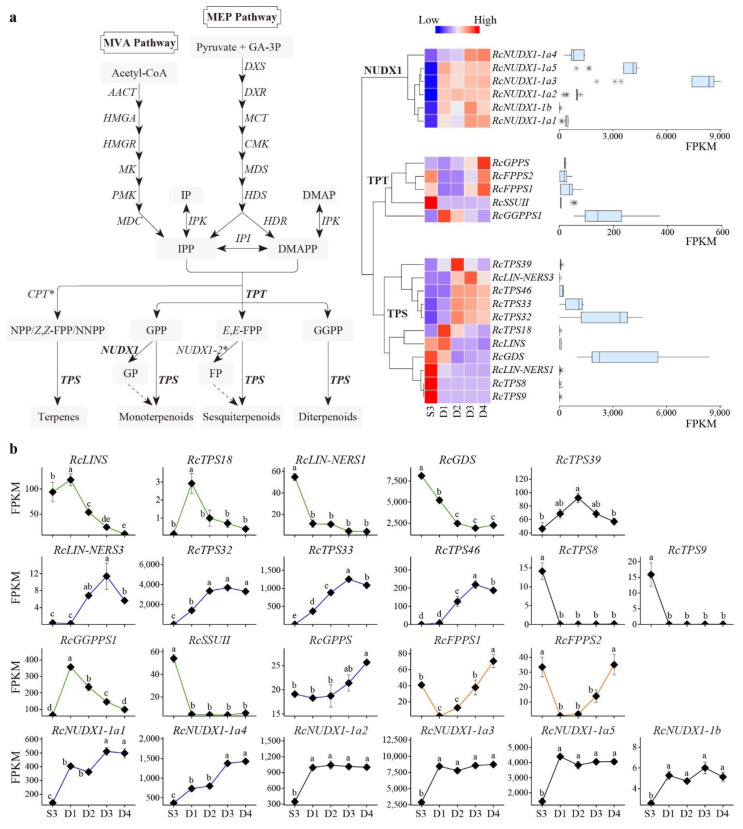
Expression profiles of *RcTPSs*, *RcTPTs*, and *RcNUDX1* genes at different flower developmental stages. (**a**) Terpene biosynthesis pathways in rose [23,46] and expression levels of *RcTPSs*, *RcTPTs*, and *RcNUDX1* genes. The genes marked with asterisk (*) indicated that the average FPKM of all members of this family was less than 1 in five MU samples. Clustering_distance_rows ‘euclidean’, clustering_method ‘complete’. (**b**) The gene expression patterns in different MU samples. Bars represent the standard error (*n* = 3). The genes with different expression patterns are illustrated in different colors. Different lowercase letters indicate statistically significant differences among samples at different developmental stages (ANOVA test, *p* < 0.05).

There are nine *RcTPS* genes belonging to three subfamilies (TPS-a, -b, and -g) expressed in MU opening petals, whereas the *RcTPS8* and *RcTPS9* expressed only in the buds about to open (S3) and hardly expressed in the petals of open flowers. These petal-expressed *RcTPSs* exhibited great differences in the expression levels and patterns (Figure 5a). *RcGDS*, which was responsible for catalyzing the synthesis of germacrene D as the only product, exhibited the highest average expression level. *RcTPS32* and *RcTPS33*, which encoded the same protein sequences, exhibited the second and third highest expression levels, respectively. The expression levels of other *RcTPSs* in petals and buds was relatively low.

The oscillations in *RcTPS* expression at different developmental stages of MU petals were analyzed. These nine genes exhibited three expression patterns. The first group comprised *RcLINS*, *RcTPS18*, *RcLIN-NERS1*, and *RcGDS*, whose expression peaked in the buds about to open (S3) or in the early opening flowers (D1) and declined in old flowers. The second group comprised *RcLIN-NERS3*, *RcTPS32*, *RcTPS33*, and *RcTPS46*, whose expression levels increased when the flowers opened and peaked in old flowers. The third group comprised *RcTPS39*, whose peak expression was on the second day after flowering (D2) (Figure 5b). These petal-expressed *RcTPSs* were selected to further validate the RNA-seq results in five samples using qRT-PCR. The results confirmed the differential TPS gene expression patterns in different samples (Figure S4). The concordance between qRT-PCR and RNA-seq results demonstrated the reliability of RNA-seq data in the present study.

### 3.6. Effect of Developmental Stages on RcTPT and RcNUDX1 Expression

The expression profiles of *RcTPTs* and *RcNUDX1s* genes were analyzed at different flower developmental stages. Among the five short-chain *RcTPT* genes involved in the biosynthesis of floral volatile terpenes, *RcGGPPS1* exhibited the highest average expression level. Similar to the expression profiles of RcTPSs, there are also three expression patterns in *RcTPTs*. The *RcGGPPS1* and *RcSSUII* exhibited highest expression in buds or fresh flowers, and *RcGPPS* expression peaked in senescent flowers, whereas the expression of two *RcFPPS* genes peaked in buds and old flowers.

There are six *RcNUDX1* genes expressed in MU petals. Among them, *RcNUDIX1-1a3* showed the highest average expression level (Figure 5a). All six *RcNUDIX1* genes exhibited significantly increased expression after flowering. Four *RcNUDIX1s* exhibited constant expression throughout the flowering stage, whereas *RcNUDIX1-1a1* and *RcNUDIX1-1a4* exhibited significantly higher expression in old flowers than in fresh flowers (Figure 5b).

### 3.7. Analysis of Differential Volatile Terpenes and Differential Gene Expression 

**Figure 6:**
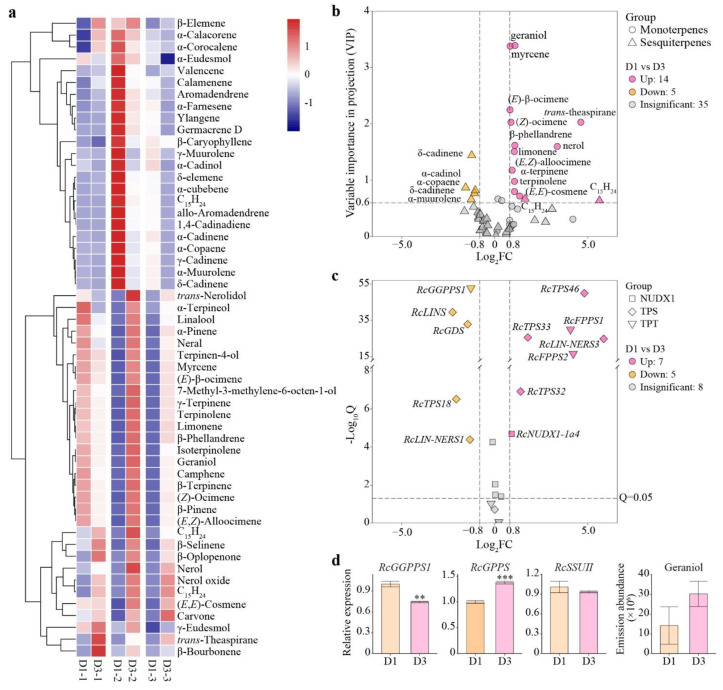
Differential metabolites and genes involved in middle and downstream pathway of volatile terpene biosynthesis in different samples. (**a**) Heatmap of volatile terpenes in D3 and D1 samples. Clustering_distance_rows ‘euclidean’, clustering_method ‘complete’. (**b**) Volcano plot of differential volatile terpenes between D1 and D3 samples. (**c**) Volcano plot of differential terpene-related genes between D1 and D3samples. (**d**) Emission abundance of geraniol and expression levels of three *RcTPTs* in D1 and D3 samples. *RhUBI2* was used as an internal control. Data are presented as the mean ± standard error (*n* = 3). Asterisks indicate significant differences between D1 and D3 samples at ** *p* < 0.01; *** *p* < 0.001 by Student’s *t*-test.

Based on the differential expression patterns of petal-expressed *RcTPSs*, the butterfly rose petals sampled on the anthesis day (D1, fresh flower) and day 3 post-anthesis (D3, old flower) were selected for volatile analysis. In total, 24 monoterpenoids and 30 sesquiterpenoids were identified (Figure 6a). The levels of almost all monoterpenes emitted from D3 samples were higher than the levels of those from D1 samples, whereas different sesquiterpenes exhibited peak release from D1 or D3 samples. As the VIP value of most volatile terpenes was very low, differential metabolites were screened based on |Log_2_FC| ≥ 0.8 and VIP ≥ 0.6. Compared with D1 samples, 14 compounds in the D3 sample including two sesquiterpenes and 12 monoterpenoids were upregulated, whereas five sesquiterpenoids were downregulated (Figure 6b).

Differential gene expression analysis between D1 and D3 samples were conducted. Differential expression of genes involved in the middle and downstream pathway of terpene biosynthesis (short-chain *RcTPTs*, *RcTPSs*, and *RcNUDX1s*) were screened based on |Log_2_FC| ≥ 0.8 and Q < 0.05 (Figure 6c). Compared with D1 samples, four *RcTPSs* and *RcNUDX1-1a4* were upregulated and four *RcTPS* genes were downregulated in D3 samples. Both *RcFPPS1* and *RcFPPS2* were upregulated in D3 samples.

The relationship between DEGs and differential volatiles was analyzed. *RcGDS* expression was higher in D1 samples than in D3 samples, which was consistent with the decreased germacrene D emission in D3 samples (40% of D1 samples). Germacrene D was not classified as a differential volatile because of its low VIP value. Increased *RcLIN-NERS3*, as well as decreased *RcLINS* and *RcLIN-NERS1*, were expressed in D3 samples, whereas the linalool emission level was higher in D3 samples (130% of D1 samples). The chirality of linalool could not be detected due to the detection method limitations. Thus, it is difficult to analyze the correlation between linalool and these three genes. 

The emission abundances of geraniol, nerol, and some monoterpenes [myrcene, (*Z*)-ocimene, and (*E*)-β-ocimene] from the D3 samples were 1.3–2.1 times those from the D1 samples, which might be related to the upregulated expression of *RcNUDX1-1a4* in D3 samples. However, the protein sequences of *RcNUDX1-1a4* was identical with other three *RcNUDX1-1* genes, so that the qRT-PCR validation of *RcNUDX1-1a4* were not carried out. In order to verify the correlation between the *RcTPT* expression levels and emission amounts of geraniol, volatile analysis and qRT-PCR were performed between D1 and D3 samples (Figure 6d). The results showed that only *RcGPPS* showed similar expression trend to emission of geraniol, suggesting that it might be related to the biosynthesis of geraniol.

### 3.8. Functional Analysis of Petal-Expressed RcTPSs in Butterfly Rose

**Figure 7:**
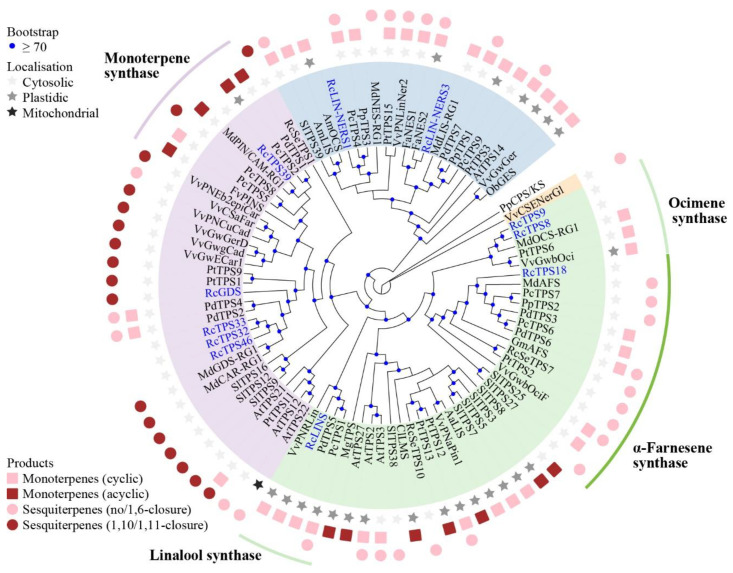
Maximum likelihood phylogeny of petal-expressed *RcTPSs* and other characterized terpene synthases. *RcTPSs* are highlighted in blue. The sequences used in this analysis are listed in Table S5. The subfamilies are illustrated with different colors: TPS-a (purple), TPS-b (green), TPS-e/f (orange), and TPS-g (blue).

Phylogenetic analysis was performed using the maximum likelihood method, including nine petal-expressed *RcTPSs*, two bud-expressed *RcTPSs,* and other characterized TPS genes (Figure 7). Of these, five *RcTPS-a* genes expressed in MU petals were grouped into two clusters. *RcTPS39* was clustered with some genes that can catalyze monoterpenoid products, such as *FvPINS* (strawberry) [45], *PcTPS2* and *PcTPS5* (*P. campanulate*) [47], *PdTPS1* (*Prunus dulcis*) [48], and *MdPIN/CAM* (*Malus domestica*) [49]. An evolutionary analysis on the TPS-a genes of Poaceae exhibited that some TPS-a members can convert GPP into monoterpenes derived from an initial C6-C1 closure [50]. Further studies will be conducted to investigate the ability of *RcTPS39* to catalyze monoterpene products. The other four *RcTPS-a* genes were mainly clustered with *TPSs* that catalyzed (*E*,*E*)-FPP to C_15_ products by an initial C10-C1 or C11-C1 closure. *RcTPS32*, *RcTPS33*, and *RcTPS46* might have the ability to convert (*E*,*E*)-FPP to produce sesquiterpenes.

**Figure 8:**
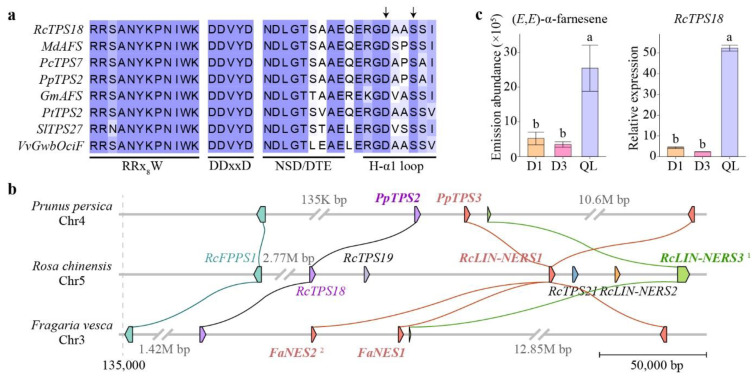
Functional analysis of *RcTPS18*. (**a**) Protein sequence alignment of *RcTPS18* and other (*E*,*E*)-α-farnesene synthases. The arrows indicated the crucial residues for AFS activity. (**b**) Micro-synteny analyses among *Rosa chinensis, Prunus persica* and *Fragaria vesca*. The collinear gene pairs are linked in the same color. The characterized genes are in bold. 1: Characterized *RcLIN-NERS3* with incomplete sequence. 2: The location of *FaNES2* in the strawberry genome was revised based on its amino acid sequence. (**c**) Emission abundance of (*E*,*E*)-α-farnesene and *RcTPS18* expression levels in three rose samples. *RhUBI2* was used as an internal control. QL: *Rosa* ‘Qinglian Xueshi’. Data are presented as the mean ± standard error (*n* = 3). Different lowercase letters indicate statistically significant differences (ANOVA test, *p* < 0.05).

The four *RcTPS-b* genes were grouped into three clusters. *RcTPS8* and *RcTPS9* were clustered with ocimene synthases, whereas *RcLINS* was clustered with other linalool synthases. *RcTPS18* was clustered together with other angiosperm α-farnesene synthases (AFSs) from *Malus domestica* (*MdAFS*) [51], *Prunus campanulate* (*PcTPS7*) [47], peach (*PpTPS2*) [52], *Glycine max* (*GmAFS*) [53], *Populus trichocarpa* (*PtTPS2*) [54], tomato (*SlTPS27*) [13], grapevine (*VvGwbOciF*) [55], and *Ricinus communis* (*RcSeTPS7*) [56], forming an α-farnesene synthase cluster (Figure 7). All genes in this cluster were predicted or identified as being localized in the cytoplasm. Sequence analysis showed that *RcTPS18* exhibited 59% to 66% identity with other AFSs of Rosaceae plants. *RcTPS18* and other AFSs exhibited conserved structural features including the RRx_8_W motif, DDxxD motif, NSD/DTE motif, and H-α1 loop. The last motif demonstrated function in the binding of the metal ion K^+^ in *MdAFS* (Figure 8a) [57].

The distribution of *RcTPS18*, *RcLIN-NERS1,* and *RcLIN-NERS3* on the chromosome is very close, forming a gene cluster (Figure 3). Interspecific micro-synteny analysis found that the chromosome distributions and functions of these three TPS gene were relatively conserved in peach and strawberry (Figure 8b). *RcLIN-NERS1* and its three collinear genes (*FaNES1*, *FaNES2*, and *PpTPS3*) showed same catalytic functions [21,45,52]. The *RcTPS18* and *PpTPS2* were colinear gene pairs, indicating that they may have similar functions.

In order to verify the correlation between the expression level of *RcTPS18* and emission amounts of (*E*,*E*)-α-farnesene, volatile analysis and qRT-PCR were performed among D1, D3 and petals of *Rosa* ‘Qinglian Xueshi’. The results showed that the emission amounts of (*E*,*E*)-α-farnesene in the three rose samples had the same trend as the *RcTPS18* expression levels (Figure 8c). A combined analysis of sequence homology, conserved structural features, volatile emissions and qRT-PCR analysis indicated that *RcTPS18* may encodes (*E*,*E*)-α-farnesene synthase.

## 4. Discussion

### 4.1. Evolution and Function of the RcTPT Genes

The present study documented that the *R*. *chinensis* genome comprised seven complete *RcTPTs*, indicating that the number of TPTs in *R*. *chinensis* is less than those reported in *Arabidopsis thaliana* (16), tomato (10), *Cinnamomum camphora* (10), and *Oryza sativa* (12), and more than or equal to that in *Chlamydomonas reinhardtii* (4) and *Physcomitrella patens* (7) (Table S5) [25,28,58]. Unlike the TPT gene family in *Cinnamomum camphora* (Lauraceae) that has segmentally duplicated TPT genes [28], no such TPT genes were observed in the genome of *R*. *chinensis*, which may be due to that *R. chinensis* exhibited only the core eudicot-specific gamma whole-genome triplication with no recent polyploidization [59].

The RcFPPS is predicted to produce FPP, the precursor of sesquiterpene, so the expression patterns of *RcFPPSs* combined the expression patterns of different putative sesquiterpene synthase genes. Both homodimeric and heterodimeric geranyl(geranyl)diphosphate synthase are involved in monoterpene biosynthesis [60,61,62]. Among the five homodimeric GPPS clustered with RcGPPS (Figure 2), three enzymes (SlDPPS, CrGPPS, and MiGPS1) were predicted to be located in mitochondria like RcGPPS. Both CrGPPS and MiGPS1 produced GPP as the sole or main product with IPP and DMAPP as substrates [60,63]. However, the SlDPPS produced C45 and C50 prenyl diphosphates [64]. The VvGPPS and AtPPPS were predicted cytosolic localization. The expression of *VvGPPS* were up-regulated preceding and during the increase in precursor volatile organic compounds of monoterpenol [65]. The AtPPPS that originally proposed as a homomeric C_10_-geranyl pyrophosphate is identified as a *trans*-type polyprenyl pyrophosphate synthase, so it is suggested that the precursor C_10_-GPP for monoterpene biosynthesis in Arabidopsis may be provided only by heteromeric G(G)PPS [66]. Although the *RcGPPS* expression and geraniol emission in MU samples showed similar trend, the products of RcGPPS in rose need further research.

### 4.2. Evolution of the TPS-b and TPS-g Genes in R. chinensis

There are four complete TPS-g genes in the genome of *R. chinensis* that were distributed in a small segment (65 kb) of the chromosome (Figure 3). The *RcLIN-NERS1* exhibited collinearity with other plants, which indicates its ancient origin (Figure 4). However, the *RcLIN-NERS1* and the other three TPS-g genes differed in gene structure (Figure S2) and were located on different phylogenetic clusters (Figure 7). Moreover, *RcLIN-NERS1* and *RcLIN-NERS3* exhibited different expression patterns during the flowering stage (Figure 5b), indicating the divergence of *RcTPS-g* gene functions.

In the RcTPS-b subfamily, both *RcTPS18* and *RcTPS-p8* had collinear TPS gene pairs in other plants (Figure 4). The *RcTPS18* was located on a special phylogenetic cluster, which differed from other petal-expressed RcTPS-b genes (Figure 7). Some TPSs in this cluster can catalyze GPP and FPP to acyclic monoterpenes or sesquiterpenes, respectively. Although many TPSs have broad substrate specificity and catalyze several substrates in vitro, their function in vivo may be limited due to their subcellular localization [67]. The *RcTPS18* protein sequence was predicted to be located in the cytoplasm. Thus, it may use FPP as a substrate to catalyze the formation of acyclic terpenoids, demonstrating that the TPS-b gene subfamily has undergone complex gene loss and duplication events [68]. *RcTPS-p8*, a putative pseudogene that encodes a short protein (455 aa), had collinear genes in all representative plants. The collinear genes of *RcTPS-p8* in *R. rugosa* (Chr6.5828, 587 aa) and peach (ONI18546, 629 aa) may be functional, but the collinear genes in grapevine (VIT_12s0059g02710, 519 aa) and strawberry (FvH4_6g43710, 144 aa) were also putative pseudogenes. Thus, *RcTPS-p8* might have lost fragments from its ancestral gene during evolution.

### 4.3. Different TPS Expression Profiles during Flower Developmental Stages

Some *RcTPS* genes exhibited peak expressions in fresh flowers or buds that are about to open. In most plants producing floral scents, volatile emission peaks when the flowers are ready for pollination and decreases afterwards [69]. Corresponding to this phenomenon, the TPS genes encoding scent biosynthetic enzymes typically peak 1–2 days ahead of emission of the corresponding compound, and the related TPS expression decreases during petal senescence stages when scent emission declines, such as the *LoTPS1* and *LoTPS2* in *Lilium* ‘Siberia’ [70,71,72]. Previous studies have reported that germacrene D emitted from the petals of *Rosa* ‘Fragrant Cloud’ reached a maximum value in mature petals and then decreased [17,20], which is similar to the *RcGDS* expression pattern observed in the present study.

Notably, some *RcTPSs* exhibited increased expression in late stage of flowers. In *Osmanthus fragrans* flowers, the expression of *OfTPS2* that exclusively produced linalool increased from the full flowering stage to the late full flowering stage [73]. Only a small amount of linalool and its oxides were released at the late full flowering stage, whereas more glycosylated linalool and its oxides were accumulated in the flower [73]. Some other plants also released or accumulated higher terpenoids in old flowers. For example, higher levels of 1,8-cineole and β-ocimene were emitted from senescent ginger (*Hedychium coronarium*) flowers, and maximum monoterpenes accumulated in old flowers of some wild *Rosa* species [24,74]. Additionally, higher levels of caryophyllene and β-cubebene were released in old flowers of *Rosa* ‘Honesty’ [19]. Since glycosylation is involved in regulating the release of volatile terpenes, the correlation between increased *TPS* gene expression and terpene emission in senescent petals needs further research.

## 5. Conclusions

In this study, we identified 7 full-length *RcTPT* genes and 49 putative functional RcTPSs in the *R*. *chinensis* genome. There are 20 genes, expressed in the opening petals of butterfly rose, were involved in middle and downstream pathway of volatile terpene biosynthesis, including 9 *RcTPS*, 5 short-chain *RcTPT*s, and 6 *RcNUDX1*. These terpene-related genes exhibited different expression patterns during five different flower developmental stages. The emissions of geraniol were higher from old MU petals than from fresh MU petals, which might be related to the *RcGPPS* expression. Combining volatile emissions, bioinformatic analysis and differential expression analysis, it is indicated that *RcTPS18*, a member of the TPS-b subfamily, may encode (*E*,*E*)-α-farnesene synthase. The highly expressed *RcTPS32*, a predicted sesquiterpene synthase, exhibited increased expression in senescent petals, deserves further study on its products and functions. The present study provided valuable insights into the terpenoid biosynthesis mechanism in rose flowers.

## Figures and Tables

**Table 1:** Members of full-length *RcTPT* genes and their sequence characteristics.

Group	Name	Id	Chr	Amino Acid	Exon Number	Localization		Conserved Motif
						TargetP	LOCALIZER	
SC ^1^	*RcGGPPS1*	RchiOBHmChr2g0102671	2	363	1	C ^3^	C	DDX_(2–4)_D, DDXXD, CXXXC
	*RcGGPPS2*	RchiOBHmChr3g0493061	3	360	1	C	C	DDX_(2–4)_D, DDXXD, CXXXC
	*RcSSUII*	RchiOBHmChr6g0279181	6	329	2	C	C	DDX_(2–4)_D, DDXXE, CXXXC, CXXXC
	*RcGPPS*	RchiOBHmChr5g0014811	5	426	12	M ^4^	M	DDX_(2–4)_D, DDXXD
	*RcFPPS1*	RchiOBHmChr5g0000321	5	342	12	/	/	DDX_(2–4)_D, DDXXD
	*RcFPPS2*	RchiOBHmChr5g0075621	5	342	11	/	/	DDX_(2–4)_D, DDXXD
LC ^2^	*RcSPPS*	RchiOBHmChr5g0028851	5	421	6	/	C	DDX_(2–4)_D, DDXXD

^1^ SC: short-chain; ^2^ LC: long-chain; ^3^ C: Chloroplast; ^4^ M: Mitochondria.

**Table 2:** Members of 49 complete RcTPS genes.

Name	Id	Chr	Sub-Family	Amino Acid	Name	Id	Chr	Sub-Family	Amino Acid
*RcTPS1*	RchiOBHmChr1g0313881	1	a	562	*RcTPS24*	RchiOBHmChr5g0023471	5	e/f	799
*RcTPS2*	RchiOBHmChr1g0326051	1	a	581	*RcTPS25*	RchiOBHmChr5g0023641	5	e/f	724
*RcTPS3*	RchiOBHmChr1g0326061	1	a	580	*RcTPS26*	RchiOBHmChr5g0036921	5	a	561
*RcTPS4*	RchiOBHmChr1g0326071	1	a	546	*RcTPS27*	RchiOBHmChr5g0037011	5	a	555
*RcTPS5*	RchiOBHmChr1g0326251	1	a	556	*RcTPS28*	RchiOBHmChr5g0037601	5	a	549
*RcTPS6*	RchiOBHmChr1g0326391	1	a	556	*RcTPS29*	RchiOBHmChr5g0038021	5	a	565
*RcTPS7*	RchiOBHmChr1g0331211	1	b	583	*RcGDS*	RchiOBHmChr5g0038101	5	a	565
*RcLINS*	RchiOBHmChr2g0160421(RcTPS-p9)+ RchiOBHmChr2g0160441(RcTPS-p10)	2	b	601	*RcTPS31*	RchiOBHmChr5g0044191	5	a	565
					*RcTPS32*	RchiOBHmChr5g0059501	5	a	557
*RcTPS8*	RchiOBHmChr2g0160561	2	b	570	*RcTPS33*	RchiOBHmChr5g0059511	5	a	557
*RcTPS9*	RchiOBHmChr2g0160591	2	b	501	*RcTPS34*	RchiOBHmChr5g0059541	5	a	544
*RcTPS10*	RchiOBHmChr2g0162311	2	e/f	852	*RcTPS35*	RchiOBHmChr5g0065101	5	a	557
*RcTPS11*	RchiOBHmChr3g0474411	3	a	560	*RcTPS36*	RchiOBHmChr6g0245751	6	a	557
*RcTPS12*	RchiOBHmChr3g0474441	3	a	560	*RcTPS37*	RchiOBHmChr6g0246001	6	a	559
*RcTPS13*	RchiOBHmChr3g0474501	3	a	560	*RcTPS38*	RchiOBHmChr6g0252721	6	b	569
*RcTPS14*	RchiOBHmChr3g0474541	3	a	557	*RcTPS39*	RchiOBHmChr6g0265741	6	a	567
*RcTPS15*	RchiOBHmChr3g0475221	3	a	560	*RcTPS40*	RchiOBHmChr6g0270581	6	a	564
*RcTPS16*	RchiOBHmChr3g0484891	3	b	566	*RcTPS41*	RchiOBHmChr6g0274871	6	a	553
*RcTPS17*	RchiOBHmChr4g0418071	4	a	564	*RcTPS42*	RchiOBHmChr6g0290871	6	c	857
*RcTPS18*	RchiOBHmChr5g0004591	5	b	580	*RcTPS43*	RchiOBHmChr6g0290941	6	c	825
*RcTPS19*	RchiOBHmChr5g0004631	5	b	583	*RcTPS44*	RchiOBHmChr6g0305391	6	a	570
*RcLIN-NERS1*	RchiOBHmChr5g0004711	5	g	544	*RcTPS45*	RchiOBHmChr7g0210371	7	a	565
*RcTPS21*	RchiOBHmChr5g0004731	5	g	509	*RcTPS46*	RchiOBHmChr7g0212441	7	a	558
*RcLIN-NERS2*	RchiOBHmChr5g0004761	5	g	580	*RcTPS47*	RchiOBHmChr7g0227831	7	a	571
*RcLIN-NERS3*	RchiOBHmChr5g0004801	5	g	580	*RcTPS48*	RchiOBHmChr7g0228501	7	a	539

## Data Availability

Not applicable.

## Supplementary Materials

The following supporting information can be downloaded at: https://www.mdpi.com/article/10.3390/genes13071177/s1, Table S1: Characterized *trans*-prenyltransferases in *Arabidopsis thaliana*, *Solanum lycopersicum* and other plants; Table S2: Primer sequences of genes used for qRT-PCR analysis; Table S3: Members of TPT-p and TPS-p genes in *Rosa chinensis*; Table S4: The corresponding IDs of *RcNUDX1* genes in *Rosa chinensis*; Table S5: Characterized terpene synthase in other plants; Table S6: Numbers of TPT subfamilies in the genomes of nine plant. Figure S1: Alignments of seven *trans*-prenyltransferases amino acid sequences in *Rosa chinensis*; Figure S2: Phylogenetic relationships (a), conserved motifs (b), and gene structure analysis (c) of the *Rosa chinensis* TPS gene family; Figure S3: Collinearity analysis of TPT genes among *Rosa chinensis* and other representative plant species; Figure S4: qRT-PCR validation of eight *RcTPSs* expressed in five samples of butterfly rose.
