# Supplementary material for: Effect of Developmental Stages on Genes Involved in Middle and Downstream Pathway of Volatile Terpene Biosynthesis in Rose Petals
Source: Genes (Basel). 2022 Jun 30;13(7):1177. doi: 10.3390/genes13071177 (PMC9320630; doi:10.3390/genes13071177)
Supplement: Supplementary file 1 [file genes-13-01177-s001.zip › genes-1739767-supplementary.pdf]

Supplementary

Table S1. Characterized *trans*-prenyltransferases in *Arabidopsis thaliana, Solanum lycopersicum* and other plants.

**Gene**	**Species**	**Genome id / accession no.**	**BLASTp**
AtFPPS2	*Arabidopsis thaliana*	AT4G17190	yes
AtFPPS1	*Arabidopsis thaliana*	AT5G47770	yes
AtPPPS2	*Arabidopsis thaliana*	AT3G20160	yes
AtPPPS	*Arabidopsis thaliana*	AT2G34630	yes
AtSPPS1	*Arabidopsis thaliana*	AT1G78510	yes
AtSPPS2	*Arabidopsis thaliana*	AT1G17050	yes
AtSSUII	*Arabidopsis thaliana*	AT4G38460	yes
AtGGPPS1	*Arabidopsis thaliana*	AT1G49530	yes
AtGGPPS2	*Arabidopsis thaliana*	AT2G18620	yes
AtGGPPS3	*Arabidopsis thaliana*	AT2G18640	yes
AtGGPPS4	*Arabidopsis thaliana*	AT2G23800	yes
AtGGPPS11	*Arabidopsis thaliana*	AT4G36810	yes
AtGFPPS1	*Arabidopsis thaliana*	AT3G14530	yes
AtGFPPS2	*Arabidopsis thaliana*	AT3G14550	yes
AtGFPPS3	*Arabidopsis thaliana*	AT3G29430	yes
AtGFPPS4	*Arabidopsis thaliana*	AT3G32040	yes
SlFPPS1	*Solanum lycopersicum*	Solyc12g015860	yes
SlDPPS	*Solanum lycopersicum*	Solyc08g023470	yes
SlSPPS	*Solanum lycopersicum*	Solyc07g061990	yes
SlSSUI	*Solanum lycopersicum*	Solyc07g064660	yes
SlSSUII	*Solanum lycopersicum*	Solyc09g008920	yes
SlGGPPS1	*Solanum lycopersicum*	Solyc11g011240	yes
SlGGPPS2	*Solanum lycopersicum*	Solyc04g079960	yes
SlGGPPS3	*Solanum lycopersicum*	Solyc02g085700	yes
SlTPT1	*Solanum lycopersicum*	Solyc02g085710	yes
SlTPT2	*Solanum lycopersicum*	Solyc02g085720	yes
VvGPPS	*Vitis vinifera*	AAR08151.1	
CrGPPS.LSU	*Catharanthus roseus*	AGL91645.1	
CrGPPS.SSU	*Catharanthus roseus*	AGL91646.1	
CrGPPS	*Catharanthus roseus*	AGL91647.1	
HlGPPS.SSU	*Humulus lupulus*	ACQ90681.1	
HlGPPS.LSU	*Humulus lupulus*	ACQ90682.1	
MpGPPS.SSU	*Mentha piperita*	AAF08792.1	
MpGPPS.LSU	*Mentha piperita*	AAF08793.1	
NtGGPPS1	*Nicotiana tobacum*	ADD49734.1	
NtGGPPS2	*Nicotiana tobacum*	ADD49735.1	
HbSPPS	*Hevea brasiliensis*	ABD92707.1	
CbGPPS.SSU	*Clarkia breweri*	AAS82870.1	
AmGPPS.SSU	*Antirrhinum majus*	AAS82859.1	
AmGPPS.LSU	*Antirrhinum majus*	AAS82860.1	
MiFPS	*Mangifera indica*	AFJ52720.1	
MiGPS1	*Mangifera indica*	AFJ52721.1	
MiGPS2	*Mangifera indica*	AFJ52722.1	

Table S2. Primer sequences of genes used for qRT-PCR analysis.

Code	Primer sequence	Gene
RcLINS-F	GGCTGAGGGTGAGAGAGGTG	*RcLINS*
RcLINS-R	AGGTTTGGCACTCTCTTGGTT	
RcTPS18-F	AGCACATGGGGAGAGAATGG	*RcTPS18*
RcTPS18-R	CTCTTGCTCAGCCGCAGAA	
RcLIN-NERS1-F	GAACTCACTCACTTCACAGCA	*RcLIN-NERS1*
RcLIN-NERS1-R	CAATCTTGCCCACGACTCTC	
RcGDS-F	TTGAGTGGATCTTCAGTGACCCTA	*RcGDS*
RcGDS-R	TTATGGTTTGTTCTTCTGTCGCAC	
RcTPS39-F	GTTTGCTACGCTATACAAGCTATGA	*RcTPS39*
RcTPS39-R	AGCAACACTCATATACTCCTCCA	
RcLIN-NERS3-F	CACAGAAGCTGTTAATAGATGGGA	*RcLIN-NERS3*
RcLIN-NERS3-R	ACCTTGTAGCTGATTTCGTTGG	
RcTPS32+33-F	TCTGGAGTTTGTCAGCGTACTT	*RcTPS32+33*
RcTPS32+33-R	GGCACGGATATCCCACCTCT	
RcTPS46-F	TCCTTCGATTGGTTGGACACT	*RcTPS46*
RcTPS46-R	GTCCTCTCTTTTCCTCAAACTTGT	
RcGGPPS1-F	ATGTGCAGTTGTTCTCGGGT	*RcGGPPS1*
RcGGPPS1-R	GTCCTTCCCAGCAGTCTTCC	
RcGPPS-F	GGGCACGTCAGCATCTCTTG	*RcGPPS*
RcGPPS-R	CCAAGGTAGTCAAGCGCAATCTC	
RcSSUII-F	CTGTGCTCTCGAAATGGTGC	*RcSSUII*
RcSSUII-F	ATTGCCATGTCTTCGCCGTA	
RhUBI2-F	TGGTTACTGCTTGCCCGAAA	*RhUBI2*
RhUBI2-R	GAAATCCGAAGTGTGCGAGC	
Actin-F	CCCTTAACCCCAAGGCCAAT	*Actin*
Actin-R	CCCAGAGTCAAGAACAATACCAGT	

Table S3. Members of TPT-p and TPS-p genes in *Rosa chinensis*.

Group	Name	Id	Chr	Strand	Subfamily	CDS (bp)	Amino acid	Exon number
TPT-p	RcTPT-p1	RchiOBHmChr1g0353891	1	-	/	393	130	1
TPT-p	RcTPT-p2	RchiOBHmChr1g0382941	1	-	/	393	130	1
TPT-p	RcTPT-p3	RchiOBHmChr2g0173741	2	-	/	393	130	1
TPT-p	RcTPT-p4	RchiOBHmChr3g0466051	3	+	/	540	179	1
TPT-p	RcTPT-p5	RchiOBHmChr5g0013331	5	-	/	171	56	1
TPT-p	RcTPT-p6	RchiOBHmChr5g0014881	5	+	/	327	108	3
TPT-p	RcTPT-p7	RchiOBHmChr5g0014891	5	+	/	528	175	4
TPT-p	RcTPT-p8	RchiOBHmChr5g0023661	5	-	/	393	130	1
TPT-p	RcTPT-p9	RchiOBHmChr5g0045111	5	+	/	393	130	1
TPT-p	RcTPT-p10	RchiOBHmChr5g0069311	5	+	/	510	169	2
TPS-p	RcTPS-p1	RchiOBHmChr1g0326011	1	+	a	1239	412	6
TPS-p	RcTPS-p2	RchiOBHmChr1g0326031	1	+	a	444	147	2
TPS-p	RcTPS-p3	RchiOBHmChr1g0326301	1	-	a	261	86	2
TPS-p	RcTPS-p4	RchiOBHmChr1g0326311	1	-	a	708	235	4
TPS-p	RcTPS-p5	RchiOBHmChr1g0326331	1	-	a	1167	388	5
TPS-p	RcTPS-p6	RchiOBHmChr1g0331201	1	+	b	867	288	5
TPS-p	RcTPS-p7	RchiOBHmChr1g0339491	1	-	a	426	141	2
TPS-p	RcTPS-p8	RchiOBHmChr2g0160361	2	+	b	1368	455	5
TPS-p	RcTPS-p11	RchiOBHmChr3g0474051	3	+	a	1098	365	4
TPS-p	RcTPS-p12	RchiOBHmChr3g0490681	3	+	a	1221	406	6
TPS-p	RcTPS-p13	RchiOBHmChr4g0406361	4	-	a	447	148	3
TPS-p	RcTPS-p14	RchiOBHmChr5g0023441	5	+	e/f	1569	522	11
TPS-p	RcTPS-p15	RchiOBHmChr5g0029171	5	+	a	666	221	3
TPS-p	RcTPS-p16	RchiOBHmChr5g0029181	5	+	b	1014	337	5
TPS-p	RcTPS-p17	RchiOBHmChr5g0029191	5	-	b	516	171	2
TPS-p	RcTPS-p18	RchiOBHmChr5g0037511	5	+	g	1185	394	4
TPS-p	RcTPS-p19	RchiOBHmChr5g0037521	5	+	g	585	194	2
TPS-p	RcTPS-p20	RchiOBHmChr5g0038061	5	-	a	210	69	2
TPS-p	RcTPS-p21	RchiOBHmChr5g0060241	5	-	a	648	215	3
TPS-p	RcTPS-p22	RchiOBHmChr5g0060261	5	-	a	918	305	5
TPS-p	RcTPS-p23	RchiOBHmChr5g0060571	5	+	a	1422	473	6
TPS-p	RcTPS-p24	RchiOBHmChr6g0245681	6	+	a	816	271	4
TPS-p	RcTPS-p25	RchiOBHmChr6g0245691	6	+	a	765	254	4
TPS-p	RcTPS-p26	RchiOBHmChr6g0246011	6	-	a	1368	455	6
TPS-p	RcTPS-p27	RchiOBHmChr6g0274881	6	-	a	396	131	2

Table S4. The corresponding IDs of *RcNUDX1* genes in *Rosa chinensis*.

Gene	Id	Description
RcNUDX1-1a1	RchiOBHmChr2g0142061+RchiOBHmChr2g0142051	Nudix hydrolase
RcNUDX1-1a2	RchiOBHmChr2g0142071	Nudix hydrolase
RcNUDX1-1a3	RchiOBHmChr2g0142081	Nudix hydrolase
RcNUDX1-1a4	RchiOBHmChr2g0142111	Nudix hydrolase
RcNUDX1-1a5	RchiOBHmChr2g0142121	Nudix hydrolase
RcNUDX1-1b	RchiOBHmChr4g0436181	Nudix hydrolase
RcNUDX1-2a	RchiOBHmChr4g0436151	Nudix hydrolase
RcNUDX1-2b	RchiOBHmChr6g0244161	Nudix hydrolase
RcNUDX1-3	RchiOBHmChr4g0436191	Nudix hydrolase

Table S5. Characterized terpene synthase in other plants.

Gene	Species	TPS Subfamily	Genome id / accession no.
AaLIS	*Artemisia annua*	b	AAF13356.1
AmLIS	*Antirrhinum majus*	g	ABR24418.1
AmOCS	*Antirrhinum majus*	g	AAO42614.1
AtTPS12	*Arabidopsis thaliana*	a	At4g13280
AtTPS14	*Arabidopsis thaliana*	g	At1g61680
AtTPS2	*Arabidopsis thaliana*	b	At4g16730
AtTPS21	*Arabidopsis thaliana*	a	At5g23960
AtTPS22	*Arabidopsis thaliana*	a	At1g33750
AtTPS27	*Arabidopsis thaliana*	b	At3g25820
AtTPS3	*Arabidopsis thaliana*	b	At4g16740
ClLMS	*Citrus limon*	b	AAM53946.1
FaNES1	*Fragaria* x *ananassa*	g	P0CV94.1
FaNES2	*Fragaria* x *ananassa*	g	P0CV95.1
FvPINS	*Fragaria vesca*	a	O23945.2
GmAFS	*Glycine max*	b	Glyma17g05500
MdAFS	*Malus domestica*	b	AAO22848.2
MdCAR	*Malus* 'Golden Delicious'	a	AGB14624.1
MdGDS	*Malus* 'Golden Delicious'	a	AGB14625.1
MdLIS	*Malus* 'Golden Delicious'	g	AGB14629.1
MdNES	*Malus* 'Golden Delicious'	g	AGB14626.1
MdOCS	*Malus* 'Golden Delicious'	b	AGB14628.1
MdPIN/CAM	*Malus* 'Golden Delicious'	a	AGB14627.1
MgTPS	*Magnolia grandiflora*	b	ACC66282.1
ObGES	*Ocimum basilicum*	g	AAR11765.1
PcTPS1	*Prunus campanulata*	b	AIC76493.2
PcTPS2	*Prunus campanulata*	a	AIC76494.2
PcTPS4	*Prunus campanulata*	g	AIC76496.1
PcTPS5	*Prunus campanulata*	a	AIC76497.1
PcTPS6	*Prunus campanulata*	b	AIC76498.1
PcTPS7	*Prunus campanulata*	b	AIC76499.1
PcTPS8	*Prunus campanulata*	a	AIC76500.1
PcTPS9	*Prunus campanulata*	g	AIC76501.1
PdTPS1	*Prunus dulcis*	a	QEE82239.1
PdTPS2	*Prunus dulcis*	a	QEE82240.1
PdTPS3	*Prunus dulcis*	b	QEE82241.1
PdTPS4	*Prunus dulcis*	a	QEE82242.1
PdTPS5	*Prunus dulcis*	b	QEE82243.1
PdTPS6	*Prunus dulcis*	b	QEE82244.1
PdTPS7	*Prunus dulcis*	g	QEE82245.1
PpCPS/KS	*Physcomitrella patens*	outgroup	BAF61135.1
PpTPS1	*Prunus persica*	g	PRUPE_4G030400
PpTPS2	*Prunus persica*	b	PRUPE_4G029900

PpTPS3	*Prunus persica*	g	PRUPE_4G030300
PtTPS1	*Populus trichocarpa*	a	AEI52901.1
PtTPS2	*Populus trichocarpa*	b	AEI52902.1
PtTPS3	*Populus trichocarpa*	g	AEI52903.1
PtTPS6	*Populus trichocarpa*	b	AII32470.1
PtTPS9	*Populus trichocarpa*	a	AII32473.1
PtTPS11	*Populus trichocarpa*	a	AII32475.1
PtTPS12	*Populus trichocarpa*	b	AII32476.1
PtTPS13	*Populus trichocarpa*	b	AII32477.1
PtTPS15	*Populus trichocarpa*	g	AII32468.1
RcSeTPS1	*Ricinus communis*	a	AEQ27766.1
RcSeTPS10	*Ricinus communis*	b	AEQ27769.1
RcSeTPS7	*Ricinus communis*	b	AEQ27768.1
SlTPS3	*Solanum lycopersicum*	b	Solyc01g105870
SlTPS5	*Solanum lycopersicum*	b	Solyc01g105890
SlTPS7	*Solanum lycopersicum*	b	Solyc01g105920
SlTPS8	*Solanum lycopersicum*	b	Solyc01g105960
SlTPS9	*Solanum lycopersicum*	a	Solyc06g059885
SlTPS12	*Solanum lycopersicum*	a	Solyc06g059930
SlTPS16	*Solanum lycopersicum*	a	Solyc07g008680+Solyc07g008690
SlTPS25	*Solanum lycopersicum*	b	Solyc02g079890
SlTPS27	*Solanum lycopersicum*	b	Solyc02g079910+Solyc00g154480
SlTPS38	*Solanum lycopersicum*	b	Solyc02g079840
SlTPS39	*Solanum lycopersicum*	g	Solyc10g005390
VvCSaFar	*Vitis vinifera*	a	ADR74198.1
VvCSENerGl	*Vitis vinifera*	e/f	ADR74219.1
VvGwbOci	*Vitis vinifera*	b	ADR74204.1
VvGwbOciF	*Vitis vinifera*	b	ADR74207.1
VvGwECar1	*Vitis vinifera*	a	ADR74192.1
VvGwgCad	*Vitis vinifera*	a	ADR74199.1
VvGwGer	*Vitis vinifera*	g	ADR74217.1
VvGwGerD	*Vitis vinifera*	a	ADR74196.1
VvPNaPin1	*Vitis vinifera*	b	ADR74202.1
VvPNCuCad	*Vitis vinifera*	a	ADR74226.1
VvPNEb2epiCar	*Vitis vinifera*	a	ADR74228.1
VvPNLinNer2	*Vitis vinifera*	g	ADR74211.1
VvPNRLin	*Vitis vinifera*	b	ADR74209.1

Table S6. Numbers of TPT subfamilies in the genomes of nine plant.

Species	TIDS-a	TIDS-b	TIDS-c	TIDS-d	TIDS-e	Sum
	FPPS	SPPS	GPPS/PPPS/ DPPS	GGPPS/GFPPS/ GGPPS.LSU	SSU	
Rosa chinensis	2	1	1	2	1	**7**
Arabidopsis thaliana	2	2	1	10	1	**16**
Solanum lycopersicum	1	1	1	5	2	**10**
Cinnamomum camphora	2	2	2	4	1	**10**
Oryza sativa	5	2	2	2	1	**12**
Picea abies	0	0	0	7	0	**7**
Physcomitrella patens	1	2	2	2	0	**7**
Selaginella moellendorffii	2	1	1	1	1	**6**
Chlamydomonas reinhardtii	1	1	1	1	0	**4**

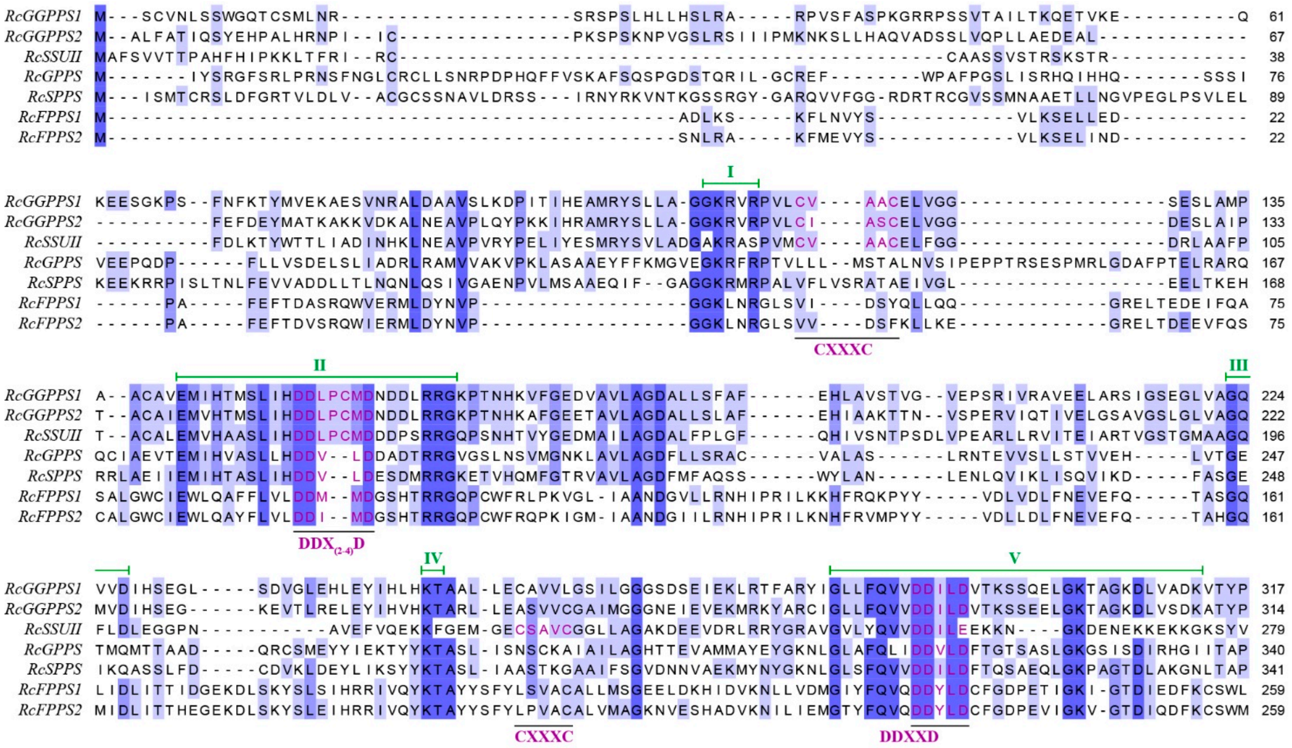

Figure S1. Alignments of seven *trans*-prenyltransferases amino acid sequences in *Rosa chinensis*. Five regions, which are conserved among the *trans*-prenyltransferases are indicated by green color.

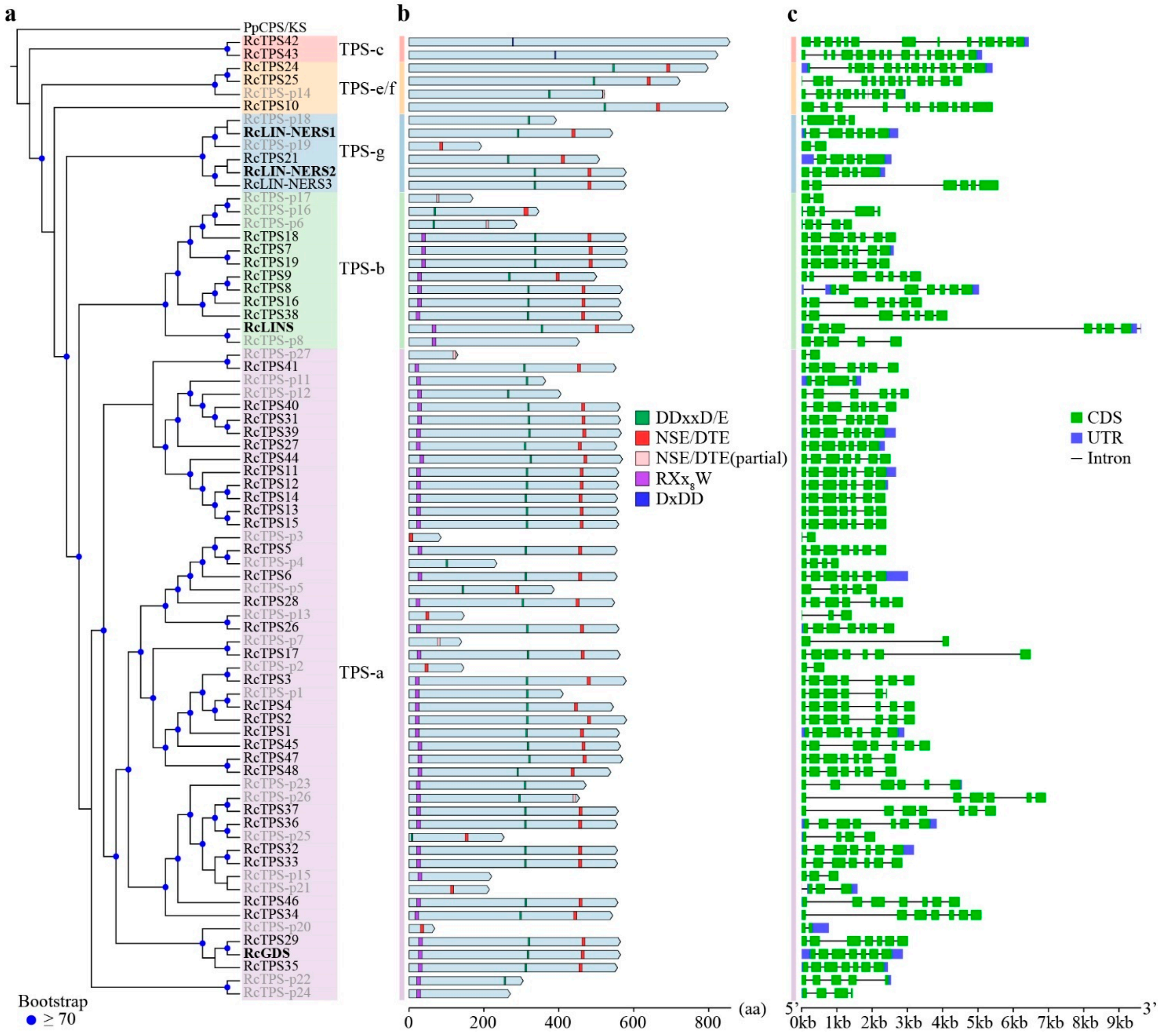

Figure S2. Phylogenetic relationships (a), conserved motifs (b), and gene structure analysis (c) of the *R. chinensis* TPS gene family. A maximum likelihood phylogenetic tree was constructed by aligning the amino acid sequences of 74 putative TPS proteins in the *R. chinensis* genome. Black letters represent putative complete TPS genes, and gray letters represent putative partial/pseudo TPS (TPS-p) genes. The characterized genes are in bold, except *RcLIN-NERS3* because its cloned sequence is incomplete. Five subfamilies are illustrated with different colors.

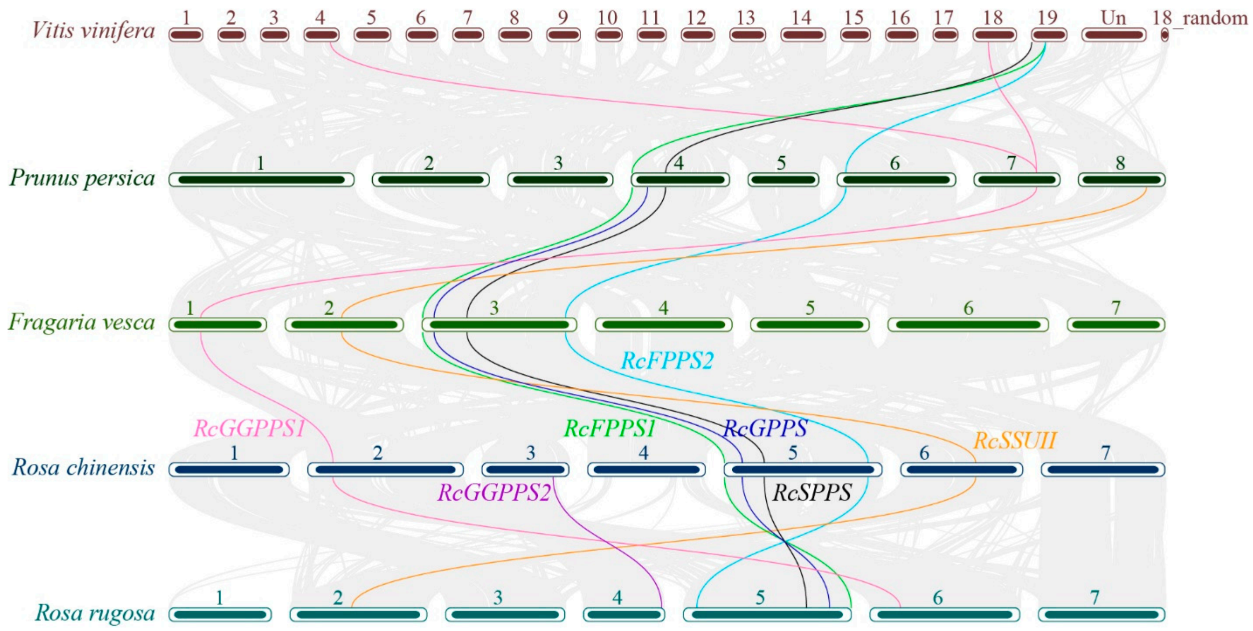

Figure S3. Collinearity analysis of TPT genes among *R. chinensis* and other representative plant species. The color and black lines highlight the syntenic TPT gene pairs. The assembly of *SSUII* gene in the grapevine genome (VIT_03s0038g03050, 1685aa) is inaccurate, so it fails to reflect its collinearity among different plants.

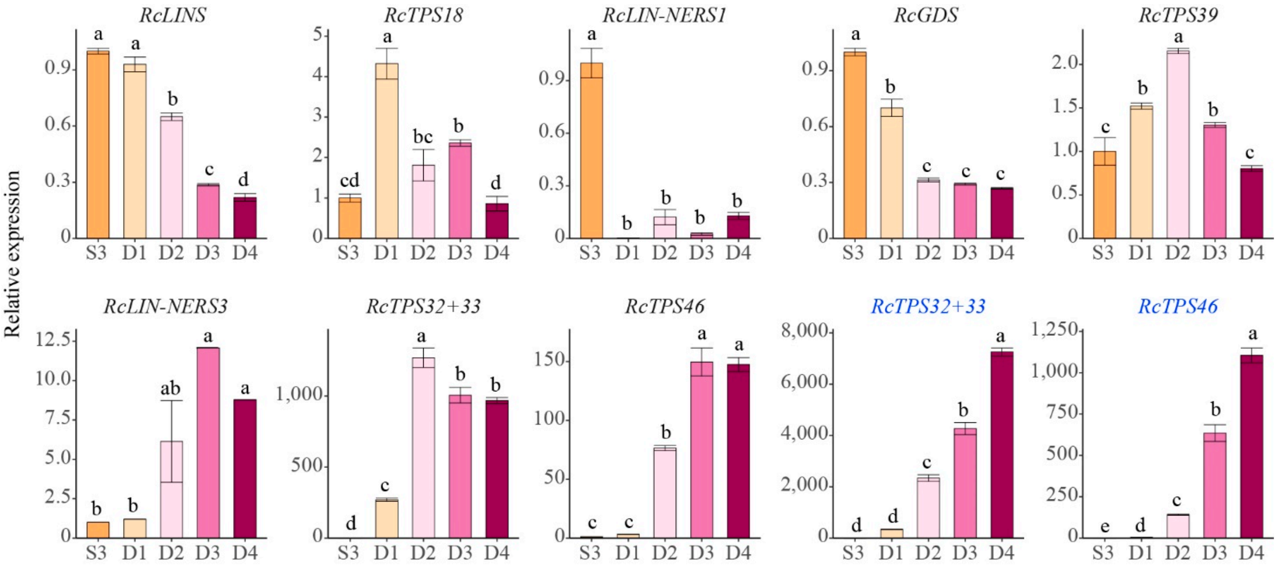

Figure S4. qRT-PCR validation of *RcTPSs* expressed in five samples of butterfly rose. Bars represent standard error ($n = 3$). Different lowercase letters indicate statistically significant differences (ANOVA test, $P < 0.05$). The genes with black names used *RhUBI2* as housekeeping gene, and the genes with blue names used *Actin* as housekeeping gene.
